# Evaluation of a Linear Measurement Tool in Virtual Reality for Assessment of Multimodality Imaging Data—A Phantom Study

**DOI:** 10.3390/jimaging8110304

**Published:** 2022-11-08

**Authors:** Natasha Stephenson, Kuberan Pushparajah, Gavin Wheeler, Shujie Deng, Julia A. Schnabel, John M. Simpson

**Affiliations:** 1 School of Biomedical Engineering and Imaging Sciences, King’s College London, London WC2R 2LS, UK; 2 Department of Congenital Heart Disease, Evelina Children’s Hospital, London SE1 7EH, UK; 3 Faculty of Informatics, Technical University of Munich, 80333 Munich, Germany; 4 Institute of Machine Learning in Biomedical Engineering, Helmholtz Centre Munich, 85764 Munich, Germany

**Keywords:** preoperative imaging, virtual reality, 3D measurement tools, echocardiography, magnetic resonance imaging, computed tomography, measurement accuracy

## Abstract

This study aimed to evaluate the accuracy and reliability of a virtual reality (VR) system line measurement tool using phantom data across three cardiac imaging modalities: three-dimensional echocardiography (3DE), computed tomography (CT) and magnetic resonance imaging (MRI). The same phantoms were also measured using industry-standard image visualisation software packages. Two participants performed blinded measurements on volume-rendered images of standard phantoms both in VR and on an industry-standard image visualisation platform. The intra- and interrater reliability of the VR measurement method was evaluated by intraclass correlation coefficient (ICC) and coefficient of variance (CV). Measurement accuracy was analysed using Bland–Altman and mean absolute percentage error (MAPE). VR measurements showed good intra- and interobserver reliability (ICC ≥ 0.99, *p* < 0.05; CV < 10%) across all imaging modalities. MAPE for VR measurements compared to ground truth were 1.6%, 1.6% and 7.7% in MRI, CT and 3DE datasets, respectively. Bland–Altman analysis demonstrated no systematic measurement bias in CT or MRI data in VR compared to ground truth. A small bias toward smaller measurements in 3DE data was seen in both VR (mean −0.52 mm [−0.16 to −0.88]) and the standard platform (mean −0.22 mm [−0.03 to −0.40]) when compared to ground truth. Limits of agreement for measurements across all modalities were similar in VR and standard software. This study has shown good measurement accuracy and reliability of VR in CT and MRI data with a higher MAPE for 3DE data. This may relate to the overall smaller measurement dimensions within the 3DE phantom. Further evaluation is required of all modalities for assessment of measurements <10 mm.

## 1. Introduction

The past 20 years have seen major advances in the management of structural and congenital heart defects, with the development of increasingly complex surgical techniques as well as the emergence of catheter-based and minimally invasive interventions. As complexity has increased, operators have become more reliant on noninvasive imaging data such as echocardiography, computed tomography (CT) and magnetic resonance imaging (MRI) to plan procedures. Traditional interrogation of such 3D datasets uses a flat screen to display either two-dimensional (2D) multiplanar reconstructions (MPR) or volume-rendered images, which simulate the appearance of depth using algorithms that generate colour and lighting effects. More recently, there has been a rising interest in novel three-dimensional (3D) imaging techniques, including augmented, mixed and virtual reality (VR), together termed ‘extended reality’ (XR). These applications enable cardiac surgeons, interventionists and cardiologists to visualise and interact with 3D imaging data in an intuitive way, giving realistic depth perception and enhanced anatomical understanding [1]. It is hoped that these benefits may lead to improved outcomes for patients with structural heart disease.

An important feature of any procedural planning tool is the ability to perform reliable measurements. While there has been a surge in the number of XR systems developed for use in cardiac patients in the past 5 years, there is a paucity of published measurement validation data [2,3,4,5,6,7,8]. Measurement accuracy is the closeness of a measured value to the true value, which can only be assessed when the actual dimension (ground truth) is known [9]. Previously, XR measurement tools have been evaluated by comparison of XR measurements to another imaging platform using anatomic data where ground truth is not known. Only two publications have compared measurements in cardiac XR systems to ground truth using phantoms; however, in both studies, only a single imaging modality was assessed [5,7]. The use of XR to plan surgical or catheter intervention must be able to measure accurately in a number of different imaging modalities used to plan such procedures.

This study aims to assess the accuracy and reliability of both VR and industry-standard “flat screen” software packages to measure phantoms of known dimensions using 3D echocardiographic, CT and MRI imaging data.

## 2. Materials and Methods

### 2.1. Phantoms

**Figure 1:**
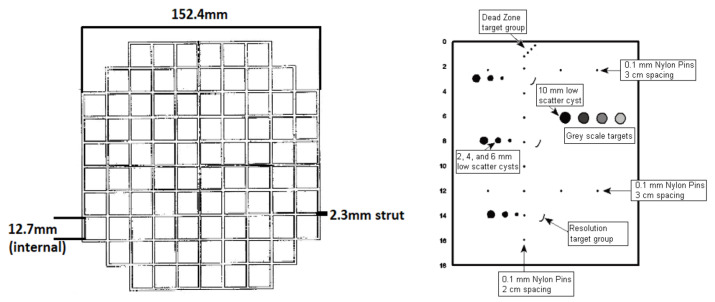
Manufacturer specifications of ACR phantom (**left**) and ultrasound phantom (**right**); images courtesy of JM Speciality Parts and Sun Nuclear.

The American College of Radiology (ACR) large head phantom was used for validation of CT and MRI measurements. It is a short hollow cylinder of acrylic plastic containing a number of internal structures designed to facilitate tests of scanner performance, including an ‘array of squares’ located within slice 5 of the standard sequence. This is a 10-by-10 array of squares with dimensions as specified by the manufacturer (JM Specialty Parts, Inc., San Diego, CA, USA) (Figure 1). These dimensions constituted the ‘ground truth’ measurements. The 403 GS LE ultrasound phantom (Sun Nuclear Corporation, Melbourne, FL, USA) was used to validate 3D echocardiographic measurements. Measurements in the ACR phantom ranged between 12.7 mm to 147.7 mm. In the 3DE phantom, measurements ranged from 6 mm to 40 mm.

### 2.2. Image Acquisition

MRI phantom images were acquired on a Magnetom Aera 1.5T (Siemens Healthcare AG, Erlangen, Germany) scanner using a 3D balanced 3D SSFP sequence with spatial resolution 1.0 mm^3^ isotropic, flip angle 90°, TE/TR 1.57/220 ms, FOV 320 × 320. CT was performed using a third-generation 192-slice dual-source scanner (Somatom Force; Siemens Healthcare AG, Erlangen, Germany) using 0.75 mm slice thickness, 512 matrix size, power 404 mA, tube voltage 140 kV. Three-dimensional echocardiographic images were obtained using a EPIQ CVx scanner (Koninklijke Philips N.V., Amsterdam, The Netherlands) as a 3D full volume using an X5 3D probe. Digital Imaging and Communications in Medicine (DICOM) files were exported to Sectra PACS (Sectra AB, Linkoping, Sweden) for CT and MRI data and TomTec Arena (TomTec Imaging Systems GmbH, Munich, Germany) for assessment.

### 2.3. Three-Dimensional Image Visualisation and Measurement

The 3D Heart VR system was created in-house using Unity (a video game development platform) with the inclusion of Insight Toolkit (ITK, a visualisation library specifically designed for scientific imaging) using a plugin system to load CT, MRI and ultrasound data [10]. DICOM files of CT and MRI studies were loaded directly into the system. Three-dimensional echocardiographic data required export to Cartesian DICOM format followed by conversion to MHD3D format using a Python script in order to be compatible with the 3D Heart system [11]. The VR software was displayed and interacted with via an HTC Vive Cosmos VR headset and controllers (HTC Corporation, Taoyuan, Taiwan). Comparative measurements were performed using the built-in line measurement tool on 3D volume-rendered images in Sectra PACS for CT and MRI images, and TomTec Arena for 3D echocardiographic data.

### 2.4. Phantom Measurement Protocol

**Figure 2:**
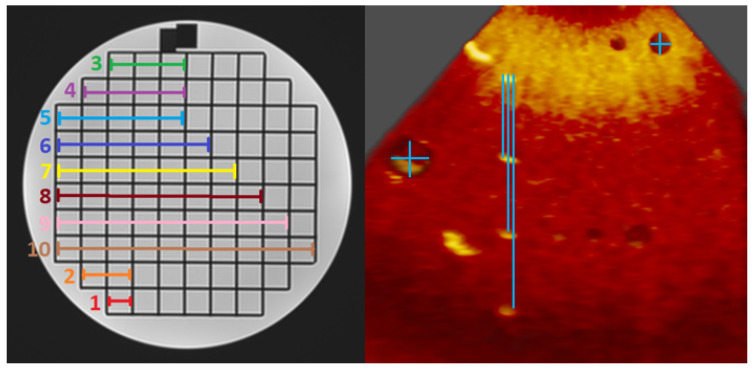
Schematic of measurements performed on CT and MRI phantom (**left**) and 3DE phantom (**right**).

Two paediatric imaging cardiologists, each with more than 10 years’ experience, performed 3 sets of 10 measurements on volume-rendered images of the MRI and CT phantom, and 3 sets of 7 measurements on the 3DE phantom (Figure 2). All measurements were performed in the 3D Heart VR system and on Sectra PACS and users were blinded to all measurements on both platforms. Identical cropping plane axes and windowing settings were provided to the participants for all measurements. The purpose of the experiment design was to ensure that visualisation of the phantom was as similar as possible between users and visualisation systems, so that measurement accuracy and precision under “ideal” circumstances was tested rather than introducing other sources of error relating to image navigation or rendering.

### 2.5. Statistical Analysis

Data analysis was performed using IBM SPSS Statistics for Windows, v27 (IBM Corp., Armonk, NY, USA). Mean absolute percentage errors (MAPE) were calculated by comparing all measurement values from both participants to ground truth values. Bland–Altman analysis was performed by calculating mean difference (‘bias’) and the limits of agreement (mean difference ±1.96 × standard deviation (SD) of mean difference), along with their 95% confidence intervals (expressed in [brackets]) [12]. Interobserver and intraobserver variability were assessed using the intraclass correlation coefficient (ICC) and the within-subject coefficient of variance (CV) [13]. ICC was calculated using the two-way mixed absolute agreement model. Significance levels were set at *p* < 0.05. CV was defined as the SD of within-subject differences expressed as a percentage of the mean [14]. All 3 repeated measurements of participant 1 were used to assess intrauser variability, and the first measurement of both participants for interuser variability. ICC values of <0.5, 0.5–0.75, 0.75–0.9 and >0.9 were regarded to reflect poor, moderate, good and excellent correlation, respectively [11]. CV <5% and 5–10% were regarded to reflect good and acceptable repeatability, respectively.

## 3. Results

A total of 162 measurements were recorded. Measures of inter- and intraobserver variability are presented in Table 1. All intraclass correlation coefficients were greater than 0.99, with *p*-values <0.001. The highest coefficients of variance for interobserver variability and intraobserver variability were in the 3DE phantom at 6% and 4.7%, respectively. When 3DE measurements less than 10 mm were excluded, CV reduced to 2.59% for interobserver variability and to 1.67% for intraobserver variability.

### 3.1. MRI

**Figure 3:**
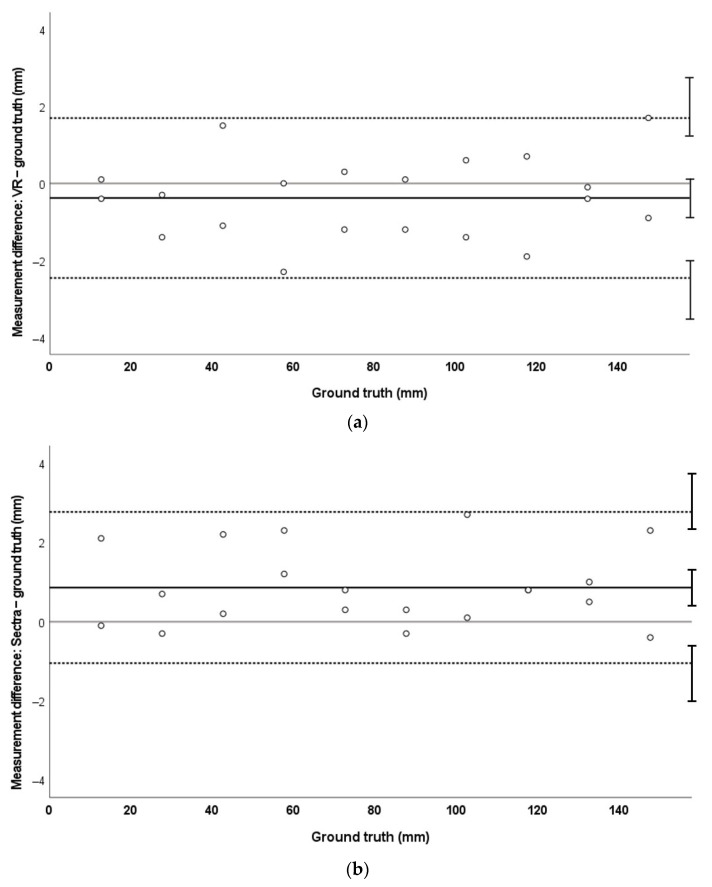
Bland–Altman plots demonstrating measurement agreement for MRI measurements against ground truth in VR (**a**) and Sectra (**b**). Mean error is represented by a solid black line, LoA by dashed lines, and 95% confidence intervals of the mean and LoA are represented by the error bars. The solid grey line signifies zero.

MAPE for measurements in VR on MRI data was 1.8%. This was compared with an MAPE of 2.4% on the industry-standard package Sectra. There was no significant bias of the VR system to over- or undermeasurement (mean of differences −0.4 mm [−0.9 to +0.1 mm]) compared to ground truth. Limits of agreement (LoA) were +1.7 mm and −2.4 mm in VR. In Sectra software, there was a bias towards overmeasurement of values (mean 0.8 mm [+0.4 to +1.3 mm]). LoA were +2.8 and −1.0 mm when compared to ground truth. These data are demonstrated in Figure 3.

### 3.2. CT

**Figure 4:**
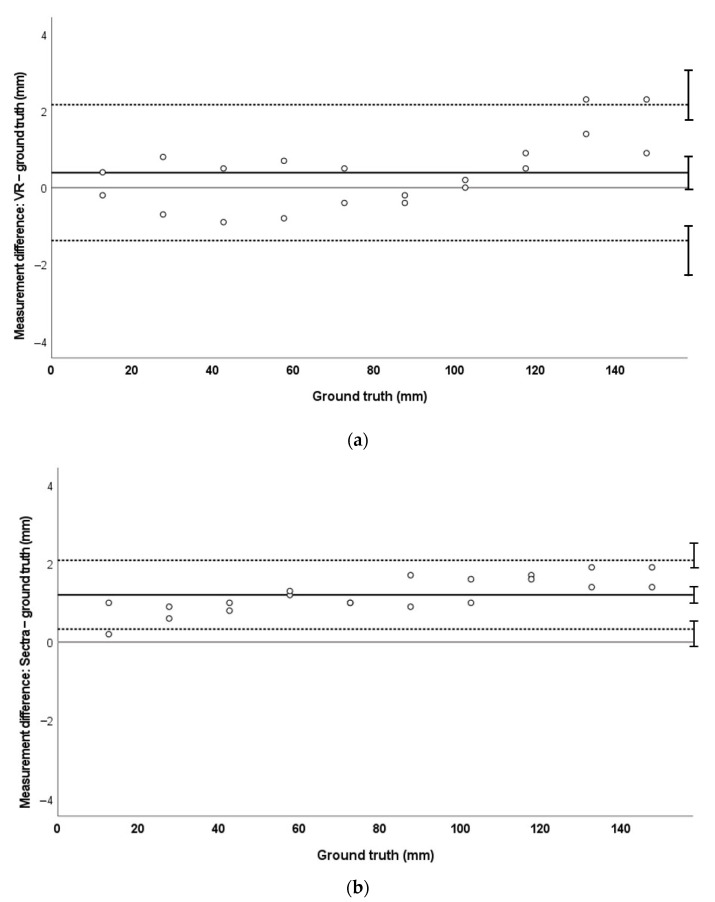
Bland–Altman plots demonstrating measurement agreement for CT measurements against ground truth in VR (**a**) and Sectra (**b**).

MAPE for CT measurements in both virtual reality and Sectra was 1.7%. There was no overall measurement bias in VR on the CT image (mean 0.4 mm [−0.03 to +0.8]) compared to ground truth. LoA in VR were −1.4 mm and +2.2 mm. For measurements performed in Sectra on the CT phantom, there was a bias towards higher values on Sectra compared to ground truth (mean +1.2 mm [+1.0 to +1.4 mm]). LoA were +0.3 mm and +2.1 mm. These data are represented in Figure 4.

### 3.3. Three-Dimensional Echocardiography

**Figure 5:**
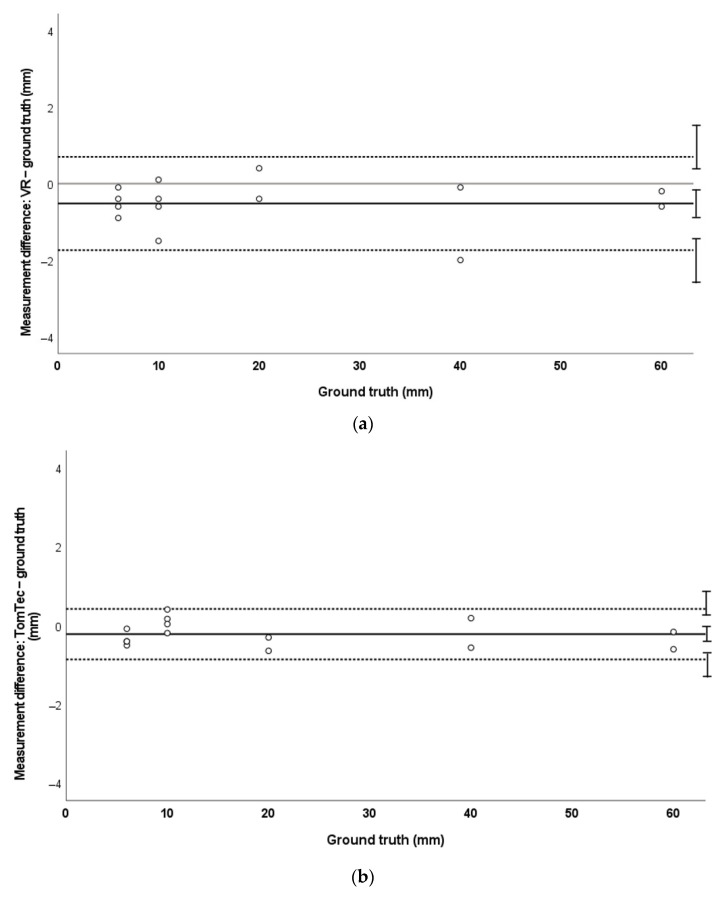
Bland–Altman plots demonstrating measurement agreement for 3D echocardiographic measurements against ground truth in VR (**a**) and TomTec (**b**).

MAPE for 3DE measurements in VR was 7.7%, compared with 2.3% in TomTec. There was a small but statistically significant bias towards smaller measurements both in VR (mean −0.52 mm [−0.16 to −0.88]) and TomTec (mean −0.22 mm [−0.03 to −0.40]) when compared to ground truth. LoA for measurements in VR were −1.7 mm to +0.7 mm. LoA for TomTec measurements were −0.9 mm and +0.4 mm. These trends are demonstrated in Figure 5.

## 4. Discussion

Intraobserver and interobserver variability, as assessed by intraclass correlation coefficient, were excellent in both VR and on standard software, with values greater than 0.99 across all imaging modalities (Table 1). When intraobserver variability was assessed by coefficient of variation, there was good agreement in all modalities in both VR and Sectra, although the value was higher in 3D echocardiographic measurements in VR at 4.7%. Interobserver variability was good for all standard software measurements and for CT and MRI in VR, and acceptable in 3DE measurements in VR at 6.01%. These results suggest that measurement reliability was lower, although still acceptable, for 3DE measurements in VR. We hypothesise that this may relate to the overall smaller measurement dimensions in the echocardiography phantom compared to those in the CT/MRI phantom, with 4/7 measurements ≤10 mm in the 3DE data compared to no measurement ≤10 mm in CT/MRI. As shown in Table 1, when the 3DE measurement values <10 mm are removed from analysis, the CV values for VR are significantly lower at 1.7% and 2.6% for intra- and interuser variability, respectively. The discrepancy in measurement dimensions between imaging modalities was a constraint of the available industry-standard imaging phantoms, which offer a limited range of measurement targets. The development of versatile cross-modality phantoms, which would facilitate the validation and calibration of existing and novel procedure planning platforms, such as extended reality, would be welcome.

Measurement accuracy showed very low MAPE for VR measurements in CT and MRI data, at 1.6% for both modalities. This was comparable with MAPE on standard software, which was 1.8% for CT and 2.2% for MRI measurements. MAPE was highest for VR measurements in the 3DE phantom at 7.7%, as compared to 2.3% in standard software. This higher error in echocardiographic data may again be explained by the significantly smaller measurement dimensions in the 3DE phantom compared to CT/MRI. MAPE magnifies differences for relatively smaller measurements; for example, a 1 mm measurement error in the 6 mm anechoic cyst would give a percentage error of 16.7%, whereas the same error in the smallest 12.7 mm measurement on the ACR phantom is 7.9%. Nevertheless, this does not explain the comparatively low error of the same measurements performed on the standard software platform. This trend may suggest lower accuracy of VR in the measurement of smaller structures. Although not performed on phantom data, studies that assessed measurements in other cardiac XR systems suggested similar or larger measurement discrepancies, especially in the smallest measurements. Sadeghi et al. reported differences between VR and 2D CT of –0.3 ± 0.9 mm and –1.4 ± 1.5 mm in measurements of paravalvar leaks [8]. Ballocca et al. compared a VR system to standard software in 3DE, with Bland–Altman plots suggesting up to 4 mm measurement discrepancy across all measurement dimensions, including those <10 mm [4].

Bland–Altman analysis demonstrated no systematic error (bias) in VR measurements in CT and MRI phantom data (Figure 3a and Figure 4a). This is in contrast to measurements made on standard clinical software (Sectra) in these data, where a systematic bias towards overmeasurement was demonstrated (Figure 3b and Figure 4b). We hypothesise that this may relate to the lack of true depth perception when viewing 3D structures on a 2D screen. In Sectra, it is possible to place measurement points at any depth in the volume-rendered image; however, perception of depth is challenging on a 2D screen and may have led to some overestimation of measurement. VR can potentially overcome these issues, as realistic depth perception and the ability to intuitively orientate and move the images can enable more confident 3D point placement. This pattern of larger-than-truth measurements was not seen in the 3DE phantom; instead, there was a small bias towards undermeasurement in both VR and the standard platform TomTec (Figure 5). However, the degree of bias was very small (VR mean −0.52 mm ± 0.36 mm; TomTec mean −0.22 mm ± 0.18 mm) and is unlikely to be of clinical significance. TomTec software differs to Sectra in that it allows users to only place measurement points on the user-defined cropping plane. While this prevents the inadvertent placement of measurements at a different depth in the image, this does not allow true perception of the 3D nature of structures, which can facilitate procedure planning.

The limits of agreement of the measurement differences were similar for VR and Sectra in MRI data (VR: −2.4 to +1.7 mm, total 4.1 mm; Sectra: −1.0 to +2.8 mm, total 3.8 mm) indicating a similar precision for both measurement tools in this modality. Limits of agreement for CT (VR −1.4 to +2.2 mm, total 3.6 mm; Sectra +0.3 to +2.1 mm, total 1.8 mm) and 3DE data (VR −1.7 to +0.7 mm, total 2.4 mm; TomTec −0.9 to +0.4 mm, total 1.3 mm) were wider in VR compared to the standard measurement tool. These results suggest lower precision of VR measurements compared to standard software. However, acceptability of a measurement tool is usually based on clinical requirements and the absolute limits of agreement were relatively small. Whether the limits determined in this experiment are significant will likely depend on the clinical situation, i.e., the overall dimensions of the structures of interest.

Performing very small measurements in VR may be more challenging for a number of reasons. On the whole, VR headsets and controllers are designed for gaming and other applications with gross controller movements, and as such, registration of very fine hand movements may not be adequately tracked and displayed in the VR space. For this study, we used the HTC Vive Cosmos headset, which uses ‘inside-out’ motion tracking, through which user and controller positions are tracked using sensors located within the headset, in contrast to traditional ‘room-space’ VR, which uses external tracking stations placed around the room. This made the VR system portable and less cumbersome, but a drawback of ‘inside-out’ motion-tracking can be lower responsiveness compared to systems using external sensors [15,16]. In addition, it may be more challenging to place measurement points in VR with very high accuracy, as controllers are held in free space without the stability provided by a desktop mouse. In future, developments in headset and tracking technology, as well as innovative mechanisms for fine point placement within the VR environment, would lend additional user confidence in this context. Further validation work is required to assess VR measurement of smaller dimensions in all modalities, and has clinical relevance for procedural planning in smaller patients and children.

### Limitations

Whilst the use of phantom data was necessary to properly assess measurement accuracy, and they are designed to simulate human tissues, they cannot substitute for the heterogeneity and complexity of real cardiac imaging data. Additionally, this study did not assess for the measurement variation, which might arise from different image acquisition techniques, such as from variation in MRI sequence parameters or other ultrasound probes. In this study, measurements were performed only on volume-rendered images, which may be more susceptible to under- or overestimation due to changes in gain or contrast than MPR; however, measurements performed within the 3D space in a user-defined fashion may be more intuitive, and arguably more useful.

## 5. Conclusions

Virtual and other forms of extended reality have potential benefits compared to traditional image visualisation software and are increasingly being used for procedural planning in patients with structural heart disease. However, data demonstrating reliability and fidelity of measurements in such systems are scarce and incomplete. To our knowledge, this is the only study that assesses measurement accuracy and reliability in the three mainstay cardiac imaging modalities in XR, using measurements of known absolute dimension as a comparison rather than another image viewing platform. This study has shown some promising data with regards to intra- and interuser variability and the overall lack of clinically significant systematic errors in VR. Overall measurement accuracy was felt to be acceptable and in keeping with other XR systems, but further work is required to further assess the performance of VR in measurement dimensions below 10 mm, which is of utmost importance when planning cases in our smallest and most vulnerable patients.

## Figures and Tables

**Table 1:** Inter- and intraobserver variability of measurements in VR and standard display.

	VR				Standard Display			
	MRI	CT	3DE (All)	3DE (>10 mm)	MRI	CT	3DE (All)	3DE (>10 mm)
Intraobserver								
ICC (95% CI)	1.00 (1.00–1.00)	1.00 (1.00–1.00)	1.00 (0.99–1.00)	1.00 (0.82–1.00)	1.00 (1.00–1.00)	1.00 (1.00–1.00)	1.00 (1.00–1.00)	1.00 (0.99–1.00)
CV (%)	1.39	1.87	4.70	1.67	1.76	1.46	1.73	0.57
Interobserver								
ICC (95% CI)	1.00 (1.00–1.00)	1.00 (0.99–1.00)	0.99 (0.99–1.00)	0.999 (0.97–1.00)	0.99 (0.99–1.00)	1.00 (0.99–1.00)	1.00 (0.99–1.00)	1.00 (0.99–1.00)
CV (%)	2.28	1.90	6.01	2.59	3.09	0.61	2.36	1.09

CV: coefficient of variance; ICC: intraclass correlation coefficient.

## Data Availability

Not applicable.
